# Medical students’ perceptions of learning and working on the COVID-19 frontlines: ‘… a confirmation that I am in the right place professionally’

**DOI:** 10.1080/10872981.2022.2082265

**Published:** 2022-05-30

**Authors:** Jennifer M. Klasen, Zoe Schoenbaechler, Bryce J. M. Bogie, Andrea Meienberg, Christian Nickel, Roland Bingisser, Kori LaDonna

**Affiliations:** a Clarunis, Department of Visceral Surgery, University Centre for Gastrointestinal and Liver Diseases, University Hospital Basel, Basel, Switzerland; b Medical University of Basel, Basel, Switzerland; c MD/PhD Program, Faculty of Medicine, University of Ottawa, Ottawa, ON, Canada; d University Hospital Basel, Basel, Switzerland, Basel; e Emergency Department, University Hospital, University of Basel, Basel, Switzerland; f Department of Innovation in Medical Education and Department of Medicine, University of Ottawa, Ottawa, ON, Canada

**Keywords:** Medical students, medical education, professional identity formation, COVID-19

## Abstract

The COVID-19 pandemic caused complex and enduring challenges for healthcare providers and medical educators. The rapid changes to the medical education landscape forced universities across the world to pause traditional medical training. In Basel, Switzerland, however, medical students had the opportunity to work on the COVID-19 frontlines. Our purpose was to understand how they perceived both learning and professional identity development in this novel context. We conducted semi-structured interviews with 21 medical students who worked in a COVID-19 testing facility at the University Hospital of Basel. Using constructivist grounded theory methodology, we collected and analyzed data iteratively using the constant comparative approach to develop codes and theoretical themes. Most participants perceived working on the pandemic frontlines as a positive learning experience, that was useful for improving their technical and communication skills. Participants particularly valued the comradery amongst all team members, perceiving that the hierarchy between faculty and students was less evident in comparison to their usual learning environments. Since medical students reported that their work on the pandemic frontlines positively affected their learning, the need to create more hands-on learning opportunities for medical students challenges curriculum developers. Medical students wish to feel like full-fledged care team members rather than observing sideliners. Performing simple clinical tasks and collaborative moments in a supportive learning environment may promote learning and professional development and should be encouraged in the post-pandemic era.

## Introduction

A growing body of literature suggests that professional identity development is both complex and dependent on a broad range of factors such as ‘socio-cultural, familial, academic, moral, religious and gender-based roles, values, beliefs and obligations’ [1]. In order to ‘think, act, and feel like a physician,’ medical students must engage in principles of identity formation including both learning experiences to gain knowledge and skills, and social interactions with patients and colleagues [2]. In medical schools around the world, however, medical students – particularly those in their first few years – may have limited, hands-on interactions with patients, and they rarely play a collaborative role on the care team [3,4].

The postponement of in-person medical education activities in response to the COVID-19 pandemic [5,6] further exacerbated these challenges, causing medical students to lose out on valuable, formative bedside teaching opportunities [7]. Although medical students have historically been sidelined during public health crises such as the 2003 SARS epidemic [8], the drastic perturbation in normal day-to-day operations caused by COVID-19 has fostered persistent concerns that medical students’ learning and professional development could suffer [9–11]. Medical students in Basel, Switzerland were, however, afforded the opportunity to work at the Triage Test Centre (TTC) – an initiative created by The Corona Task Force (CTF) of the University Hospital of Basel (UHB) to relieve some of the healthcare system pressures caused by a high flow of incoming patients and a corresponding shortage of personnel and resources [12]. Medical students were recruited to join a specialized response team called the ‘SWAB team’ [13] where, under appropriate safety protocols, they supported senior health care workers by assisting in the clinical evaluations of hundreds of patients per day.

Although observation is a useful pedagogical activity for medical students [14,15], opportunities such as medical student-run health clinics with numerous patient interactions provide an invaluable, but relatively rare educational opportunity for junior learners [16,17]. Medical students desire more hands-on learning opportunities and physicians – particularly those tasked with preparing trainees to become competent health care professionals – are constantly striving to improve the quality of education and care. Since extraordinary circumstances might reveal insights useful for transforming teaching and practice, we explored the experiences of medical students who worked at the TTC within a learning environment that was necessitated by a once-in-a-generation crisis. By examining their perspectives, we sought to identify the features of this novel setting that medical students perceived as particularly influential for skill acquisition and professional identity development.

## Methods

We conducted semi-structured interviews with medical students who volunteered in the TTC during the first wave of the COVID-19 pandemic to explore their perceptions of learning and working on the frontlines. Our iterative data collection and analysis process was informed by Constructivist Grounded Theory (CGT) [18], and all study procedures adhered to the Declaration of Helsinki and the Ethics Commission Northwest and Central Switzerland (EKNZ, Req-2021-00518).

### Study context

**Figure 1.**
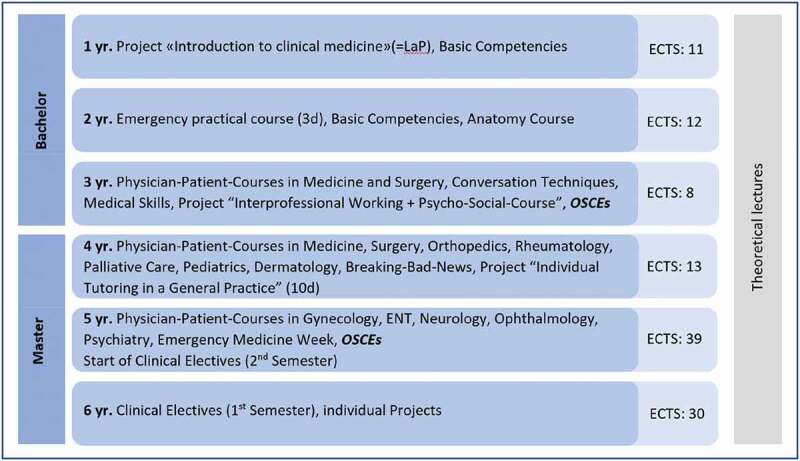
Clinical and hands-on courses at the university of Basel.

In Basel, the traditional curriculum integrates some but not routinely hands-on practices beginning in year one (Figure 1).

OSCEs = Objective Structured Clinical Examination. ENT = Ear, Nose, Throat. ETCS = European Credit Transfer and Accumulation System, which is used in most European Universities. 1 ECTS = 25 h of work.

Medical students gain their first clinical experiences through a format called Learning on the project (LaP) in the first year. LaP includes several bedside-teaching sessions and other learning opportunities in the hospital such as observing surgery. Before or during the first year, the University also expects a month-long clerkship as a nurse assistant in a hospital. In the second year, medical students work three days in the ED, guided by nurses. From the third year to the fifth year, Basel’s medical students participate in the ‘physician-patient-courses’ to learn conducting a medical history and physical exams. Those small group learning sessions cover some of the most critical fields in medicine, closely supervised. In the fourth year, medical students observe a family doctor to collect further professional insights. From the fifth to sixth year, medical students work as full-time clerks in the hospital for one year. As the final exams of the third and fifth year, OSCEs are conducted to monitor their clinical competence skills and development.

The TTC afforded medical students an experience vastly different from the traditional curriculum. The Preachers ‘ Church, where the TTC was located, operated from March to June 2020. The TTC was staffed with physicians, nurses, other healthcare providers, army staff, and medical students who worked on the ‘SWAB team’. Two shifts were scheduled per day consisting of 12 medical students each. This schedule translated to a total of 936 shifts that were covered by medical students in March and April 2020. All medical students received a paid working agreement and appropriate insurance coverage. The SWAB team curriculum included: history taking; testing with swabs; learning about social distancing and disinfection; and hygiene, self‐protection, and careful handling and donning of PPE. This curriculum was provided by experienced physicians who acted as clinical supervisors on-site. There were no formal learning objectives associated with this clinical placement, nor were there any formal examinations required of the medical students.

### Study participants and recruitment

We purposively sampled participants through both word-of-mouth communication and through the online TTC chat group. Because they had worked at the TTC from its inception and had previous bedside training experiences, we initially sampled medical students in years 4–6 of the traditional Basel curriculum (see Figure 1). These participants then recommended other possible interview candidates, so we modified our recruitment efforts to include a snowball sampling approach. As the iterative data generation and analysis process unfolded, we also theoretically sampled participants to ensure diversity in both gender and level of training, ceasing recruitment when we reached theoretical sufficiency [19]. Of the Twenty-three students who were invited to participate, two declined because of time constraints. Participation was voluntary and we obtained written informed consent before each interview.

### Data collection and analysis

As a voluntary student on the COVID-19 frontline, ZS was interested in understanding how her peers experienced learning in this novel setting. JK and ZS developed the semi-structured interview guide in Swiss German, the native language of all participants. Regarding the wording, we adjusted the interview language according to the participants as ZS interviewed them as a peer to guarantee the interviews were conversational. The interview started with an easing-in strategy, with ZS asking about students’ professional experiences in the medical field and their motivation to study medicine. ZS integrated probing questions throughout each interview to encourage participants to describe their experiences and perceptions in greater detail. For instance, ZS asked questions such as ‘What was a typical working day like?’ and “What are examples of things you learned at TTC? ‘How did the learning that occurred at TTC differ from what you learned in medical school before the pandemic?’

In the beginning, ZS asked about their work schedule and their experiences at the TTC with peers, supervisors, and patients. As the study progressed, the interview guide was extended to include questions about specific learning and working experiences as a team member at the TTC such as ‘What was your most formative experience as a team member’? as well as questions about professional identity development such as ‘What did you get from working on the Task Force for your studies and your (personal and professional) development?’

ZS conducted the interviews from May to October 2020. The interviews were held via Zoom (*n* = 17), Skype (*n* = 1), telephone (*n* = 2), and in-person (*n* = 1) using PPE and in accordance with public health guidelines. The interviews lasted between 30 and 70 minutes and were audio-recorded in Swiss German, and then transcribed and translated to German by ZS. All identifying information was removed from the transcripts prior to analysis. Using a constant comparative approach, we identified and refined codes and themes by analyzing data both within and across transcripts [18,20]. Specifically, ZS read all the transcripts, while JK read the first seven in full. During frequent meetings, ZS and JK discussed the data and developed initial, focused, and theoretical codes [18]. To support the evolving analysis, team members (JK, AM, BB, and KAL) reviewed rich transcripts and data excerpts selected by ZS and JK; of note, ZS and JK translated the representative selections of findings into English for KAL and BB to review. Quirkos Version 2.3.1 was used to manage the data. ZS wrote field notes and analytical memos that were instrumental for informing the analysis.

### Research team

ZS is a medical student at the UHB who worked at the TTC. Influenced by her experiences, ZS conducted her master’s thesis on learning and working amidst the COVID-19 pandemic. ZS’ written reflections prior to the study supported reflexivity, helping her recognize personal beliefs and acknowledge potential influences. JK, the principal investigator of the study, is a senior surgeon at the UHB who is interested in medical education research. JK incorporated her experiences and reflections as both a former medical student, resident, and current qualitative, medical education researcher and clinician teacher to guide her contributions to this study. AM, CN, and RB were involved in study design, and manuscript preparation, drawing on their knowledge and involvement in the development and management of the TTC. BB is a Canadian MD/PhD student with experiences working as a medical student on the frontlines of the pandemic in Canada. He has a diverse background in medical education research. He and KAL – a PhD trained scientist with expertise in qualitative methodologies – provided an international perspective on the current research, drawing on their experiences in the Canadian context to highlight structural differences in both traditional undergraduate medical education (UGME) curricula and medical students’ learning opportunities during COVID-19. The team acknowledges that the personal and professional disruptions they experienced because of the pandemic likely influenced their interpretations of the data.

## Results

The demographic characteristics of the 21 participants are presented in Table 1. As shown, our sample comprised a diverse group of participants according to sex, age, and stage of training. The medical students in years 4–6 (*n* = 12) worked for approximately three to four months in the Preacher’s Church TTC, while the medical students in years 1–3 (*n* = 9) worked there for approximately one to two months. The frequency of workdays varied between one to six days a week, although most of the participants worked at the TTC three days per week.

**Table 1.** Demographic characteristics of the participants

Demographic Characteristics	
Sex	*n* (%)
Male	9 (43%)
Female	12 (57%)
Experience/Year of the Medical Curriculum	
1 year	2 (10%)
2 years	2 (10%)
3 years	4 (19%)
4 years	6 (28%)
5 years	0 (0%)
6 years	7 (33%)
Age	
20	1 (5%)
22	1 (5%)
23	6 (28%)
24	3 (14%)
25	5 (24%)
26	5 (24%)

**Figure 2.**
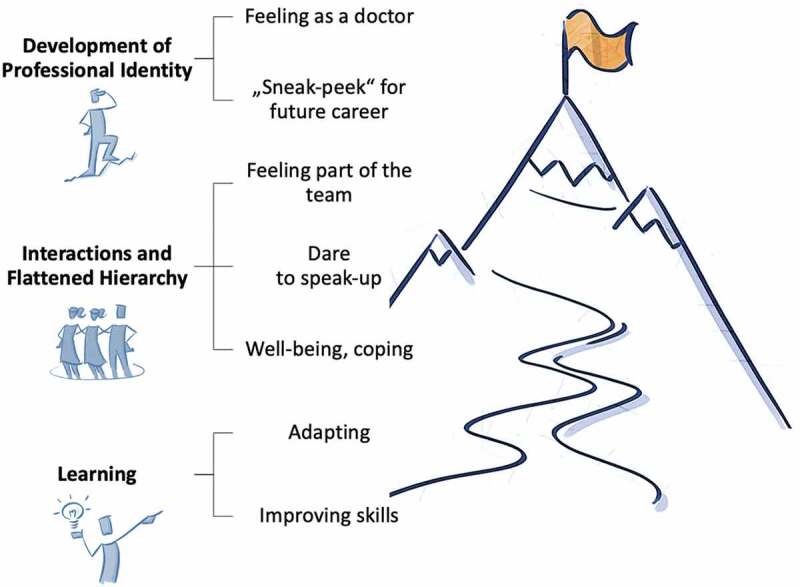
Main themes of the analysis.

Based on the data, we developed several themes illustrated in Figure 2. Focusing on our initial research aim – that is, how medical students experienced the TTC as a novel clinical learning environment and how it might inform their professional development and the post-pandemic medical education – we present the following main themes in this paper [1]: learning, adapting, and improving skills [2]; Interactions and flattened hierarchy; and [3] development of professional identity. Each theme is supported with quotes from our participants (P1-21) for illustration.

### Learning, adapting, and improving skills

The learning environment at the TTC did not look or feel like a typical clinical setting:

*‘For me, the atmosphere and mood in Preacher’s Church were exceptional. It is wonderful to see the cathedral with the pipe organs upstairs and then the people below who look like martians in their white space suits’* (P1). Working under strict hygiene provisions was also a novel feature that seemed exciting – even other-worldly – for some participants: ‘*I was strangely dressed like that because I had never worn such complete protective equipment before. Somehow you felt mega special’* (P5).

Medical students across all years of training had to quickly adapt, not only to these startling surroundings, but also to engaging autonomously in both simple and complex clinical tasks. Some tasks, such as ‘*taking the swabs*’ (P1) were generally perceived as easy to learn yet educationally impactful. Additionally, because they were given the opportunity to practice concepts they had only ever understood theoretically, even seemingly mundane tasks were perceived as extremely valuable. For example, although all medical students are taught the importance of hygiene early in their training, following proper hygiene techniques such as disinfecting hands and surfaces seemed to crystalize learning: *‘It certainly made me think about hygiene, how to properly dress and undress, what steps and in such a way that it really makes sense’* (P4).

Not surprisingly, this type of learning was particularly valuable for the more junior participants in years 1–4, who reported that the opportunity to engage in frequent hands-on patient care drastically improved their diagnostic and technical skills. For instance, medical students reported learning how to identify and consider differential diagnoses for COVID-19: *‘ … in this sense, tonsillitis, bronchitis, pneumonia, or pulmonary embolism. And yet, every now and then, a suspicion of angina pectoris’* (P21). From their patient encounters, others learned the importance of not presuming a diagnosis too early:

*Another crucial experience was when …one day someone with lung cancer and someone with temporal arteritis were discovered by students, and I found that very impressive. There is another view … if something strikes you as suspicious, you look it up again* (P7).

For the medical students in year 6, tasks such as taking nasopharyngeal swabs quickly became rote. However, although they perceived that they did not learn much that was new, their overall experience at the TTC reinforced previous learning and permitted them ample opportunities to practice various skills (P6, P7, P9). Indeed, all participants appreciated that, during busy days at the TTC when there were many incoming patients to consult, they were afforded considerable time and autonomy to improve their history taking, diagnostic, and technical skillsets. For instance, one participant remarked that working in a high-acuity setting under extraordinary circumstances helped them learn *‘ … how to organize a history taking efficiently and in a structured manner … in the end, it was … a matter of 5 minutes. And not necessarily worse, you just knew what the crucial questions were’* (P8).

Perceived improvements in clinical skills resulted not only from directly performing these tasks, but also from engaging in observational learning, such as listening to physicians and more senior medical students interact with patients: *‘What’s more … not active, but passive, if you listen to certain physicians doing triage, I find it incredibly instructive’* (P21). Another student shared that: *‘ … listening to others taking a medical history, those are things that I have to master at some point, and just as they approach the situation and talk to patients’* (P5).

Indeed, for most of the participants, the greatest perceived improvement was not in the realm of technical skills, but rather in the development of so-called ‘soft skills’ like patient-centered communication: *‘At times it was just the knowledge of human nature or dealing with patients. I think that was almost the biggest point from a medical point of view’* (P11). Every participant reported improvements in their level of communication skills and their ability to provide individual care for patients: *‘ … precisely this routine and the feeling of how to approach people and deal with them … you explain it step by step – … you meet a relatively large number of people and then have to assess how to approach them and explain everything quickly.’* (P2). Another participant described: *‘That you had to do something that was just not pleasant and then pack it in a communicative way so that it was still okay for the patient’* (P3). Some participants reported that these experiences boosted their confidence in managing difficult patients, while others reported that they developed better techniques for obtaining critical information faster while still making the patient feel understood – a skill they perceived as invaluable for their future clinical practice (P3, P21).

### Interactions and flattened hierarchy

Mostly, participants appreciated ‘ *… all the interaction with colleagues*’ (P4), noting that feeling like an integral part of the team was the most striking and valuable feature of this learning environment. For participants, performing an essential role alongside peers, physicians, nurses, and military service members – rather than observing from the wings – was both atypical and formative: ‘*we really saw in this Church that it doesn’t matter whether you are a student, a resident, or a consultant, everyone can contribute and then a team works’* (P15). For participants, this sense of comradery broke down the hierarchical barriers governing their typical interactions with faculty in traditional learning settings: *‘Especially at the beginning in the Church, it was mega cool how flat the hierarchy was, and somehow everyone communicated fully openly and yes … it was just an enjoyable working environment’* (P17). Another participant agreed that they *‘had the feeling that the hierarchy in the Preacher’s Church was lifted’* (P15). Not only did participants report that *‘it was cool to talk to the doctors’* (P13) and *‘it was always easy to talk to all physicians and yes, it was fun too’* (P8), many participants appreciated that the physicians made purposeful efforts to interact with them to promote their training: *‘I also think that the doctors did it very well and took enough time … they said: “Look, we have time, we don’t have to do … ” and then they asked about our medical knowledge, which was fun’* (P8). The TTC learning environment even emboldened some participants to speak up or pushback, with one participant finding it remarkable not only *‘ … that you dared to speak up and even criticize junior and senior consultants’* (P4), but also that:

*… other physicians were always delighted to receive feedback, like: ‘Hey, something is not going well there at the moment, can’t you try to change something?’ … And many were really grateful for it, and that was nice … And that helped me a bit to notice that most of them are satisfied when you address the problem* (P4).

While all participants obtained valuable learning, not all perceived that this experience was necessarily ‘worth it’:

*I would say that working at Preacher’s Church has undoubtedly brought me something for my studies. Still, to be honest, it would have been better for me if the COVID-19 pandemic had not broken out and we had continued our studies regularly* (P1).

However, most participants described the interactions as an opportunity to leave the house, meet others, and find the meaning of their work. Overall, most students understood the work at the TTC as a means to cope and foster their well-being during an extraordinarily stressful time.

### Development of professional identity

For most participants, their experiences at the TTC were greatly influential for both facilitating their learning *and* charting their future career paths. For instance, some described that their work on the COVID-19 frontlines provided a ‘sneak-peek’ into emergency or disaster medicine, piquing their interest in pursuing these specialities down the road (P2, P13, P17):

*I found it very exciting, and I could imagine going in this direction at some point later, in emergency medicine or something else, where something is always going on. I could still imagine that, and I don’t know whether I could have said that beforehand* (P17).

However, for most participants, their work on the frontlines validated their choice to become a doctor, noting that their work in the TTC provided ‘*such a confirmation that I am in the right place professionally’* (P21). Another participant confirmed: *‘It rather encouraged me that I think I will have a cool job that will give me back what I am looking for’* (P6). One participant neatly encapsulated the experience that all participants conveyed in various ways – working on the COVID-19 frontlines was invaluable because, for the very first time, they felt like *‘patients saw [them] as a real doctor’* (P16).

## Discussion

This study sought to investigate how medical students perceived learning and working on the frontlines during the COVID-19 pandemic. All participants reported that their experiences at the TTC were positive and effective in promoting knowledge and developing task-oriented and intrinsic skills such as communication. These experiences further promoted the professional development of the medical students during a time of uncertainty about their current and future medical education and clinical experiences.

The discrepancy of perceived learning value among participants in various training years was likely a product of the traditional curriculum structure where junior learners not routinely experience opportunities to perform tasks directly and with autonomy, nor do they have the opportunity to routinely observe physicians and senior medical students perform duties in real clinical settings [21–23].

For all learners, however, the perception that they were engaging as service-providers *not* as learners [24,25] was instrumental for their professional identity development. In other words, participants felt, often for the first time, that they were providing an essential service – a rarity in the traditional curricula, particularly for junior medical students. Features of the TTC appear to align with the upper stages of the Bates’ hierarchy of contextual competence [26]: Legitimacy and belonging in a novel healthcare setting, competence, including scope and responsibility of the learners, and autonomy [26]. Traditionally, medical students are presented with a stricter learning environment correlated with a more explicit and fixed hierarchy than that of the TTC environment [27–30]. During regular bedside teaching sessions, medical students are guided by supervisors and have a clear schedule and defined tasks to complete when interacting with patients. At the TTC, however, participants reported that the traditional hierarchy in pre-pandemic clinical environments collapsed into a perceived ‘flatter hierarchy’ wherein the medical students perceived that they were viewed more as colleagues than learners.

In our study, perhaps the widespread uncertainty about the novel coronavirus, along with the common purpose of combatting COVID-19, fostered a shared sense of learning and adaptation amongst faculty and medical students. Even if the hierarchy was not actually as flat as they perceived, perceptions matter. And the perception of being a legitimate team member both fostered confidence and reinforced participants’ sense that they had indeed pursued the right professional path. In turn, medical students interacted with physicians and other medical staff differently than they usually would in the hospital setting, adopting a more casual style, and in some cases, pushing back and offering criticism. Interacting meaningfully as part of a team seemed vital for learning and professional identity development, supporting that ‘a reciprocal and reinforcing relationship between student experiences of professional inclusivity and social exclusivity that creates a defined sense of professional identity [31].’

The TTC allowed junior learners to gain early exposure to a context of the clinical environment, translating into perceived improvements in clinical knowledge and skills, which led to competence and, with time, to autonomy – an esteemed sociocultural value in medicine rarely afforded to medical students, particularly those early in training [32] This sense of identity transformation from learner to healthcare provider is similar to the natural identity transformation that occurs when a medical student transitions from pre-clinical to clinical training [33,34]. At the TTC, this transition seemed to happen much faster for junior medical students than the traditional curriculum usually affords. Such a transition is indeed associated with increases in clinical responsibilities as medical students become more widely integrated into the clinical setting and care team. In the traditional curriculum, this transition is likely not associated with a complete flattening of the hierarchy, but more of a reduction in the natural hierarchy given the continued presence of fixed roles between medical teachers and learners. Under the unprecedented circumstances of a pandemic, learners had the opportunity to feel a sense of being a valued member of the team – providing a crucial service during a time of considerable uncertainty, which might influence the phenomenon of identity development and transformation from learner to healthcare professional.

Since learning and professional identity development depend on active engagement [35], we and others call for medical teachers, clinical supervisors, and curriculum developers to more meaningfully embed opportunities for medical students to interact with patients, to autonomously perform tasks appropriate for their level of training, and to take part in team meetings at all points during their undergraduate medical training [36,37]. While we acknowledge that the re-design of a curriculum might take a lot of creativity, the effort may pay dividends. One crucial component might be to strengthen the capability of learners to ‘engage with an uncertain and unfamiliar context in a meaningful way’ in experiencing different learning situations in the pre- and post-clinical curriculum as already recommended by Fraser and Greenhalgh [38]. Offering different learning experiences in clinical settings such as the hospital ward, emergency department, or private practices might foster the recognition to adapt to the novelty [39]. At the same time, educators have to facilitate explicit conversations about such capability development processes. Overall, the TTC appeared to accomplish these desired outcomes, suggesting that, if such in-person, interactive learning is achievable amidst a catastrophic healthcare crisis, it could feasibly become part of the ‘new normal’ of post-pandemic medical education.

## Limitations

This study reports the perspectives of medical students who consented to be interviewed about their experiences working in a novel learning environment. Findings may not necessarily reflect the perspectives of all the medical students who worked at the TTC. As a medical student herself, ZS conducted all interviews to engage her peers in a casual but candid discussion about this topic. Yet, we acknowledge that some participants may have avoided vulnerable issues regarding this unprecedented time of the pandemic. Finally, the interviews were held during the first part of a continuously evolving pandemic. Undoubtedly, this on-going crisis will continue to generate novel adaptations to learning and care that require further investigation.

## Conclusions

While most medical students around the globe continued their training from behind a computer screen, the learners at the TTC were fostering their clinical prowess in an in-person environment that allowed them to *feel* like legitimate healthcare providers. Thus, for participants, working on the COVID-19 frontlines was hugely influential for both their learning and professional identity development. Our results reiterate the need to create learning environments where medical students are afforded ample opportunities to both participate in hands-on learning *and* to feel like a valued member of the care team. Our participants told us that such opportunities do not have to be extraordinary or overly complex. That is, for medical students who primarily observe rather than play an active role in traditional clinical settings, even rudimentary (yet critically important) tasks were perceived as exciting and valuable for learning. We remind practicing physicians to remember how impactful simple clinical tasks and collaborative moments are for professional development – particularly for learning skills like communication that can be challenging to acquire and finesse without direct engagement.
